# Stigma experience and coping strategies in stroke survivors: a qualitative study

**DOI:** 10.3389/fpsyg.2025.1581639

**Published:** 2025-06-18

**Authors:** Lina Song, Xinbo Sun, Chengxia Li, Bing Li, Lijie Jing, Xuebing Jing

**Affiliations:** Zibo Central Hospital, Zibo, China

**Keywords:** stroke, stigma, cognitive regulation, nursing, qualitative study

## Abstract

**Aim:**

To investigate the true experiences of stigma and changes in stroke survivors and explore how they manage their symptoms.

**Background:**

Stroke is a serious disease that threatens human health with increasing mortality and disability rates. Declining self-care ability and excessive external dependence can easily lead to stigma. However, there is a lack of studies on real stigma experiences and coping styles among stroke survivors.

**Design:**

A descriptive qualitative study.

**Methods:**

Fourteen participants were recruited across inpatient stroke settings in China. Semi-structured face-to-face interviews were conducted with participants to collect data. Audio-recorded data were transcribed. The data were analyzed using the seven-step Colaizzi method for phenomenological analysis, adhering to the principles of Phenomenological research methodology. The study adheres to SRQR EQUATOR checklist.

**Findings:**

Fourteen semi-structured interviews were conducted, revealing three main themes and ten sub-themes: (1) Non-adaptive emotion regulation in response to stigma, including sub-themes of remorse, shame, sadness, perceived disaster, depression, and reduced self-worth; (2) Adaptive emotion regulation in response to stigma, including positive reappraisal, positive adjustment, acceptance, and support systems; (3) Origins of stigma, including sources such as relatives, friends, oneself, and medical staff.

**Conclusion:**

The findings have the potential to inform the development and implementation of strategies to reduce the experience of stigma in early-stage clinical settings. Medical professionals must prioritize the comprehensive examination of genuine instances of stigma encountered by stroke survivors. Timely identification of stigma is imperative to mitigate the risk of patients adopting inaccurate beliefs and maladaptive coping mechanisms post-stroke. Strategies aimed at diminishing stigma should consider personal, familial, policy-related, societal, institutional, and environmental dimensions.

## 1 Introduction

In China, cerebrovascular disease, recognized as a major health threat, has garnered significant attention from the Chinese government. Since the onset of the 21st century, driven by the proactive initiatives of the Stroke Prevention and Control Engineering Committee, notable advancements have been made within China’s healthcare system in the realm of stroke prevention and treatment (Chao et al., 2019). Yet, the vast demographic base in China has presented obstacles to the advancement of these efforts. Despite the ongoing progress in medical capabilities within China, the incidence and mortality rates of stroke patients continue to escalate. According to the China Stroke Prevention and Treatment Report (2023), the number of stroke patients aged 40 and above in China has reached 12.42 million, with patients skewing towards younger age groups. On average, an individual experiences either an initial or recurrent stroke every 10 s, with one person succumbing to stroke-related complications every 28 s. Among survivors, approximately 75% suffer from sequelae, with 40% experiencing severe disability.

Stroke patients are often accompanied by symptoms of nerve damage such as hemiplegia and aphasia, among which hemiplegia is the main manifestation of stroke sequelae (Chen et al., 2022). With the establishment and improvement of dedicated stroke care pathways across various regions, coupled with advancements in emergency thrombolysis and thrombectomy technologies, the mortality rate associated with stroke has seen a decline. Nevertheless, the rate of disability remains notably high (Mohamad et al., 2023). The annual disability rate of new stroke patients in China has reached as high as 75%, among which 70 to 80% still suffer from limb dysfunction after treatment (Chen et al., 2022). Post-stroke dysfunction often leads to enduring physical, psychological, cognitive, and social impairments, marked by resistance to treatment and unfavorable prognoses. Movement, language, swallowing, and emotional disruptions escalate patients’ reliance on external assistance or aids, fostering a sense of stigma (Liu et al., 2023), and predisposing them to post-stroke depression (PSD) (Di et al., 2019) and Poststroke emotionalism (PSE) (Broomfield et al., 2022), thereby heightening mortality risks. Therefore, it is imperative to focus on patients’ encounters with stigma and implement health behavior interventions accordingly. This approach holds profound importance in managing their emotional well-being and enhancing their overall prognosis.

The word “stigma,” originating from Greek and translated into English as “shame and stigma,” denotes a trait deemed immoral and detrimental to an individual’s reputation, serving as a symbolic imposition by external societal forces. Sociologist Goffman (1963) expressed the belief that stigma refers to the shame experience caused by a patient’s own performance or the negative and distorted views and attitudes of others towards a specific characteristic of the patient after the disease, which leads to the reduction of the patient’s reputation or status in the eyes of others. Link and Phelan (2001) stated that stigma was not only caused by individuals, but also by socialization. Stigma is commonly associated with specific negative or undesirable traits or characteristics, leading to the labeling of individuals with insult and contempt when possessing these attributes. Such “labels” instill attitudes of guilt, shame, and disgust in individuals, fostering experiences of discrimination, social isolation, personal harm, and a decline in social standing. According to WHO, stigma is a “hidden burden of disease,” including perception, expectation, internalization and experience of stigma. Research on stigma and neurological diseases has focused on patients with epilepsy and mental disorders (Sarfo et al., 2017), and there is a scarcity of studies on stroke patients. Compared with AIDS and cancer patients, the external characteristics of stroke patients are more obvious, and the level of stigma is also different. Disablism’ is thus added to the likes of sexism, racism, ageism and homophobia as a form of exclusionary and oppressive practice. Scambler (2009) defined stigma as an ontological deficit, reflecting infringements against norms of shame, discrimination by others on grounds of being imperfect.

Stigma can seriously affect the social function of stroke patients, precipitating negative psychological states, particularly evident in early-stage stroke patients. This may result in delays in the rehabilitation process and diminish the overall quality of life. Studies have shown that 50% of stroke patients within 1 year after the onset experience stigma problems (Sjögren, 1982). The emergence of stigma hampers the motivation and functional recovery of stroke patients during the rehabilitation process, dampening patients’ enthusiasm to engage in social activities. Stigma could potentially contribute to the development of depression following a stroke (Zhu et al., 2019). Stroke patients often struggle to cope with substantial changes brought about by the illness and are at increased risk of experiencing stigma following a cerebral infarction (Mohamad et al., 2023). The neurological impairments that persist after a stroke, combined with societal attitudes towards individuals with disabilities, frequently evoke negative emotions in patients. This can lead to self-stigmatization, where patients internalize negative societal perceptions, resulting in changes in cognition, behavior, and mood (Wang et al., 2019). Emotion-regulation strategies across psychopathology (Aldao et al., 2010) explains that people can flexibly adopt various emotional regulation strategies according to different environments to achieve the best emotional regulation effect. Cognitive emotion regulation can explain some mental symptoms, and has a certain effect on empathy disorder and stigma. Cserép et al. (2022) found that people with higher stigma were more likely to use negative emotional regulation strategies. As such, it is crucial to clinically address strategies for effectively reducing the occurrence of stigma following a stroke. The aim of the present study was to interview stroke survivors to gain insight into their firsthand experiences and subsequent changes, thereby exploring approaches to managing their symptoms.

### 1.1 What does this paper contribute to the wider global clinical community?

In addition to focusing on patients’ physical treatment, it is essential to prioritize their psychological well-being. Proactively identifying factors contributing to poor rehabilitation compliance and low mood is imperative. By doing so, the quality of patient care can be enhanced, while supporting the holistic recovery process.

The results highlight the factors contributing to patients’ experience of stigma and illuminate key areas for reducing stigma and facilitating improved recovery in the future.

Identification and coping against stigma should be established as early as possible to increase protective factors in stroke rehabilitation.

## 2 Method

### 2.1 Design

A phenomenological research approach was employed in the present study. A series of semi-structured interviews were conducted. Semi-structured in-depth interviews were combined with open-ended questionnaires. The open-ended questions were designed to provide participants with the chance to share what mattered most to them, while accompanying prompt questions were formulated to explore various themes from diverse perspectives. The in-depth interview method involved face-to-face interactions between the researcher and participants, typically lasting up to 60 min, utilizing verbal communication and non-verbal cues to facilitate discussion (Dietrich et al., 2020).

In order to ensure the originality and diversity of the questionnaire results, inter-participant communication was avoided. In the present study, participants were encouraged to articulate their stigma around stroke patients, and research and recommendations were made based on the results of the conversation. The inherent complexity and challenges of this approach help to enhance its persuasiveness and robustness (Magaldi and Berler, 2020).

### 2.2 Setting and participants

Participants were purposefully selected for the present study, recruited from neurology wards within a tertiary hospital in Zibo City, China, spanning from November 2023 to February 2024. Inclusion criteria consisted of: (1) meeting the stroke diagnosis criteria outlined in the “Neurology” journal (2018), with all participants experiencing their first onset and maintaining stability, (2) exhibiting an Energy scale (ADL) score > 40 upon admission, (3) having a National Institutes of Health Stroke Scale (NIHSS) score ≤15, (4) being aged 18 years or older, (5) possessing complete clinical data, and (6) lacking significant communication impairments. Exclusion criteria encompassed: (1) patients with cognitive or communication disorders,(2) patients with conditions known to elicit stigma, such as mental illness, HIV/AIDS, or hepatitis B, and (3) individuals with other critical illnesses like malignancy, heart failure, liver or renal failure. All participants provided informed consent before participating in the study. Initially, 11 patients were interviewed, with an additional 3 patients interviewed thereafter until saturation of new information was attained.

### 2.3 Data collection

Based on previous research (Kallio et al., 2016), a preliminary interview guide was developed to align with the study objectives. Purposive sampling was employed, and face-to-face interviews were conducted. Pre-interviews were piloted with 5 patients. The interview guide underwent review by 6 stroke specialists to ensure relevance and comprehensiveness. It comprised five open-ended questions aimed at eliciting comprehensive responses from participants. Interviews were conducted from November 2023 to February 2024. The interviewer, a female neurosurgery specialist nurse with a Master’s degree in nursing and 3 months of qualitative research training, was tasked with conducting the interviews. Interview times and locations were communicated in advance, with the patient ward chosen as the interview venue. No additional personnel were present during the interviews. Prior to each interview, patients were positioned comfortably and ensured to receive their regular treatment. Each interview, lasting 30–60 min, commenced with an introduction, and the entire conversation was audio-recorded with the participants’ consent. Techniques such as active listening, clarification, and feedback were utilized to foster open dialogue. While the interview guide provided a framework, flexibility was maintained to adapt the sequence and format of questions to delve deeper into participants’ narratives. Emotional changes, gestures, and language cues were observed and documented throughout the interviews. The final three interviews were conducted until the moderators collectively agreed that saturation had been achieved, indicating no new information was forthcoming. To avoid interviewer bias, before the study, we conducted self-reflection and position statements to avoid potential biased positions. We also carried out pre-interviews and training, enabling researchers to identify their leading questions or non-verbal biases (such as nodding to acknowledge), and adjust interview techniques, such as avoiding leading questions and reducing the likelihood of preset answers. During interviews, we maintained a non-judgmental attitude, used neutral language, and avoided showing agreement or disagreement with participants; responses, for example, by repeating confirmations (What you just said… is that right?) rather than direct evaluations. Regular reflexive journals were maintained during data collection to record how our perspectives might influence interactions with participants.

The formal outline was as follows:

- Can you tell us how the onset of the disease occurred?
- How did you react and feel after the onset of the disease?
- How did the attitudes of people around you change after the onset of the disease? What changes do you care more about?
- When you were diagnosed, did you experience any discrimination?
- How to respond when you feel any experience of discrimination?

### 2.4 Data analysis

Following each interview, relevant data were transcribed promptly within 24 h by two researchers. Interview notes were meticulously compiled, encompassing verbal expressions, eye contact, voice tone, and body language of the participants. These observations were transcribed into written text. Subsequently, the researchers cross-verified the transcriptions with the interviewees for accuracy. To analyze the qualitative data, the Colaizzi phenomenological data analysis method was employed (Liu, 2019; Su et al., 2024). Specific methods: familiarize yourself with the interview content; extract statements relevant to the study; code the recurring views; summarize and refine the codes; the views are grouped into thematic prototypes; the prototypes are defined and described; similar prototypes and descriptions are repeatedly compared, similar views are identified and extracted, and the themes are constructed; the structure of the themes is returned to the research participants for verification. This method facilitated the transition from transcription to translation, aiding in uncovering underlying themes and patterns within the data.

## 3 Findings

A total of 14 participants were interviewed in this study. For anonymity, participants are denoted as sample *N* (number). The sample comprised five males (45.5%) and six females (54.5%), with ages ranging from 39 to 73 years (mean age of 59 years). A detailed description of the participants is presented in Table 1.

**Table 1:** Description of participants.

N.	Sex	Age	Profession	Medical insurance type	Dysfunction	Previous history	Caregiver
N1	M	57	Businessman	Urban dweller	Right lower limb hemiplegic	No	Partner
N2	F	67	Nurse	Urban workers	Left lower limb hemiplegia	Glycuresis	Partner
N3	F	62	Unemployed	Urban dweller	Left side hemiplegia	Glycuresis	Partner
N4	F	63	Retired	Urban workers	Left lower limb hemiplegia	Hypertension, coronary disease, glycuresis	Partner
N5	M	39	Driver	Urban dweller	Left side hemiplegia	Hypertension	Partner
N6	F	72	Retired	Urban workers	Right lower limb hemiplegic	Hypertension	Offspring
N7	M	49	Doctor	Urban workers	Right lower limb hemiplegic	Hypertension	Partner
N8	F	70	Farmer	No	Left lower limb hemiplegia	Glycuresis	Partner
N9	M	50	Businessman	New rural cooperative	Left side hemiplegia	No	Partner
N10	M	60	Worker	New rural cooperative	Left lower limb hemiplegia	Hypertension	Partner
N11	F	61	Farmer	New rural cooperative	Incontinentia urinae	Coronary disease	Offspring
N12	M	56	Cadre	Urban workers	Right lower limb hemiplegic	Hypertension	Partner
N13	M	50	Cadre	Urban workers	Right lower limb hemiplegic	Hypertension	Partner
N14	M	47	Businessman	Urban workers	Left side hemiplegia	Hypertension	Partner

Semi-structured, in-depth interviews were individually administered to participants, revealing a notable surplus of trust placed in the researchers, surpassing initial expectations. Participants anticipated a receptive audience for their narratives. The analysis delineated three principal themes and ten subordinate themes: (1) maladaptive emotional regulation linked with feelings of stigma; (2) adaptive emotional regulation associated with feelings of stigma; and (3) origins of stigma. These thematic constructs, along with their corresponding sub-themes, are listed in Table 2.

**Table 2:** Themes and subthemes.

Theme	Subtheme
Non-adaptive emotion regulation of sickness shame	Self-blame, shame, sadness
	Catastrophizing
	Frustration
	Decreased sense of self-worth
Adaptive emotional regulation of sickness stigma	Positive Reassessment
	Active adjustment
	Acceptance
	Support System
Sources of sickness stigma	Relatives, friends, myself
	Health care workers

### 3.1 Theme 1: non-adaptive emotion regulation feelings of illness stigma

The majority of stroke survivors conveyed an inability to acknowledge their post-stroke health condition. They initiated a process of introspection regarding their lifestyle habits that may have precipitated the onset of illness, engendering feelings of panic. The repercussions of the illness on their future prospects heightened the participants’ adherence to medical recommendations. Nevertheless, this emotional response often precipitated self-blame among participants, intensifying their sense of shame. Several elderly participants even perceived themselves as burdensome to their families, with sighing becoming a commonplace expression of their distress.

Throughout the interviews, it was observed that certain respondents suffered from illnesses attributed to entrenched unhealthy lifestyle habits. These habits prove challenging to modify as they stem primarily from the individuals themselves, evoking feelings of sadness and guilt.

*“I prefer fatty meat over lean meat, and I eat a lot every day, so it’s impossible for me not to get sick. “(Participant N5). “My children advised me against consistently consuming vegetable soup and leftover meals, but I disregarded their advice. As expected, my blood sugar and blood pressure became unmanageable, I had a cerebral infarction. I feel ashamed of disregarding my children’s concern.” (Participant N3). “Despite working in the hospital for over 20 years, I neglected my own health, hypertension resulted in a cerebral hemorrhage.” (Participant N7).”I have been working in an administrative position for more than two years. Although I used to be a doctor, I rarely pay attention to my blood pressure in daily life. This incident has made me realize that no matter how busy I am, I should monitor my blood pressure, blood sugar, and blood fat.”(Participant N13).*

Many patients perceived stroke as a significant burden, a seemingly insurmountable ailment, and a catastrophic event.

*“When I was first diagnosed, I felt a strong sense of distress. Every day, I couldn’t help but think that everyone is healthy, contrasting with my own affliction” (Participant N2). “My neighbor developed this ailment, exhibiting unsteady gait and reliance on numerous medications. Whenever I envision myself in a similar state, I feel as though the world is collapsing around me” (Participant N5). “The daily expenses for treatment are substantial, compounded by my partner’s illness, and I didn’t have insurance, which added to my financial burden” (Participant N8).”I work in an office, and over the past two years, there have been others like me who have stepped back from the front line. I am the only one who has experienced a stroke.” (Participant N12).*

Many individuals believe that post-illness, numerous tasks become unattainable, and the recovery process, reliant on medication, necessitates time, leading to a perception of suffering and profound frustration.

*“I am so young, my daughter just was admitted to graduate school this year, my family need me, how can I get this disease” (Participant N1). “I am finished, my head is buzzing every day, I have been in the hospital for so long with injections, but I am not well, I am finished, my disease is hopeless” (Participant N4). “A few years ago, because of coronary heart disease, I spend a lot of money! When I see my wet bed, I feel uncomfortable, and I want to die” (Participant N11).*

The interviewees believed that they considered themselves as a useless person, a burden to others, disrespected, and had no value.

*“At the beginning, when my legs were uncomfortable, I thought it was a lumbar spine issue, but I didn’t expect that I could be incapacitated by cerebral thrombosis” (Participant N1). “I came to help my children, and now I had to let them take care of me. I’m really useless” (Participant N3). “The children are all at school, and my wife takes care of the family full-time. I am sick, and my family has no financial resources” (Participant N9). “The disease made my clothes constantly damp and the children didn’t come to see me. I do not know why I’m alive” (Participant N11).*

### 3.2 Theme 2: adaptive emotional regulation of feelings of shame

Negative emotions such as negativity, sadness, and shame significantly contribute to the participants’ heightened sense of sickness-related shame. However, through appropriate guidance, patients can expedite their acceptance of their condition and confront the impacts of the disease with a more positive outlook. Researchers observed a gradual increase in participants’ confidence in their lives as they progressed through treatment. Moreover, some older participants identified their children as a motivating factor in their journey towards recovery.

After experiencing the onset of illness, respondents may initially grapple with denial, remorse, and guilt. However, by confronting the reality of their condition and actively seeking the root causes of their predicament, they can engage in positive self-reflection. Through this process, they can address their shortcomings and limitations so as to better adjust the discomfort caused by the disease. As such, they exhibit a more confident and positive mood, enhancing their self-efficacy.

*“No one’s life is devoid of challenges, and mine is no exception. Compared to the children who lived in the hematology department, my situation is not the direst. I am determined to improve my health, and my patients are counting on me” (Participant N7). “Instead of allowing others to ridicule me, I choose to confront challenges head-on. From now on, I will prioritize self-care and lead a fulfilling life. My wife and children depend on me, and I am committed to supporting them” (Participant N9).”I must hasten my recovery. My daughter takes care of two children by herself. It is imperative that I aid my daughter and alleviate her burdens” (Participant N3).*

Most respondents underwent profound reflection following the adverse events, extracting valuable lessons to preempt similar occurrences in the future.

*“We have to try to adjust, melancholy is just one day, and happiness is another. Since God has granted me this life, I should strive for happiness and let my friends know that I am actively working on improving myself” (Participant N5). “My current task is to adhere to medication schedules, collaborate with colleagues in the rehabilitation department to ensure a successful recovery, and ultimately aim to return to my professional duties” (Participant N7).*

“Acceptance” serves as a method for patients to alleviate negative emotions, representing an adaptive strategy within cognitive emotion regulation. Through acceptance, individuals can embrace the present moment and cultivate a forward-looking perspective on life.

*“The doctor indicated that I exhibited minimal symptoms, so I slowly accepted my illness, and I realized that the most important thing is to be alive, and I looked forward positively “(Participant N1). “Observing myself walk steadily fills me with genuine happiness, knowing that my patients await my return and that I continue to contribute meaningfully” (Participant N7). “I run my own business, my wife is having her fourth child, I already have three sons, and my relatives are working with me. If I fail, my entire family will be affected” (Participant N14).*

When patients perceive greater support from their families, friends, and society, their confidence to overcome the disease strengthens. Hence, they can confront the negative impact of the disease with greater resilience, effectively minimizing its adverse effects.

*“My children send me encouraging videos daily, reminding me of my continued importance to them. With their support, I’ve learned that having the disease is not daunting; rather, my main task is to face it correctly and learn to accept it” (Participant N2). “As a veteran, I’ve faced many battles, but the country provides us with great care, and my two sensible children support me every step of the way. For their sake, I must persevere” (Participant N6). “Now, with improved conditions, I can get drugs with higher cost-effectiveness at the hospital, which is both effective and affordable, with significant medical insurance reimbursement. I am deeply grateful, as this alleviates the burden on my children” (Participant N3). “Indeed, I was fortunate. Following my illness, my relatives and friends were present to care for me. Additionally, my family’s support was very strong.” (Participant N14).*

### 3.3 Theme 3: a source of illness stigma

Stroke often results in a variety of limb-related effects, prompting participants to intricately describe the behaviors and speech patterns of those around them post-stroke. Participants exhibited a tendency to swiftly grasp, magnify, and occasionally misinterpret the perceptions of others, often without awareness. As such, this phenomenon led to a gradual increase in the participant’s sense of shame. Such negative emotional experiences profoundly impede the recovery of social roles.

*“I am overweight, and I notice my son’s discomfort when changing diapers. Additionally, I sense that my relatives avoid visiting me, possibly due to their aversion to the smell of my bed” (Participant N11). “The doctors emphasize the positive progress of my brain’s recovery, but I struggle with the prospect of facing people outside, particularly while walking with a limp. I wish the doctors would provide more psychological comfort and encouragement” (Participant N3).*

Medical staff, in particular, should maintain careful attention to their words and actions in their professional duties. Patients experiencing heightened feelings of shame are particularly susceptible to being emotionally “hurt” by the actions of their caregivers.

*“The nurses are always rushed when attending to me, quickly changing my IV bag and departing, leaving me feeling like an invalid” (Participant N9). “The other day, I overheard a nurse commenting on my young age and brain infarction, suggesting returning home to teach her husband a lesson and make him remember “(Participant N5).*

## 4 Discussion

### 4.1 Correct understanding of the sources of stigma

The stigma experienced by stroke patients is often self-imposed. Research indicates that the majority of stroke survivors suffer from some form of sequelae, with approximately 70% experiencing physical dysfunction to varying extents. Many patients harbor fears of societal discrimination due to these sequelae, such as hemiplegia and urinary incontinence, as well as the resulting burden on their families. These concerns lead to avoidance of social interactions, self-perceived social isolation, and a profound sense of illness stigma. Disability is not just a medical condition but also a social phenomenon, often accompanied by societal prejudice against individuals with disabilities. According to a survey study on stroke conducted in the Western context (Sarfo et al., 2017), approximately 80% of patients reported experiencing mild to moderate levels of illness stigma. Additionally, 14.5% of participants felt personally responsible for their stroke, while 13% expressed embarrassment regarding their physical limitations. The development of illness-related shame due to their own unhealthy lifestyle habits aligns with the findings of Yu et al. (2016), who observed that smoking, a common poor lifestyle habit, serves as a source of shame for patients with lung cancer.

Illness-related shame can be instigated by the actions and perceptions of relatives, friends, and the general public (Nejatisafa et al., 2017). Blame from close relations and companions can exacerbate feelings of self-blame among patients, contributing to an intrinsic sense of sickness shame. Further, research (Yu et al., 2016) suggests that a lack of family support intensifies a patient’s perception of sickness shame. Stroke patients, predominantly aged 60–69 years and comprising more men than women, often feel limited in their ability to contribute to society and family, potentially leading to negative treatment by their families (Hu, 2020). Such attitudes can foster a sense of sickness stigma among elderly patients (Anderson and Whitfield, 2013). It is essential to recognize that medical staff, aside from providing treatment and care, patients should be informed about the cause, pathogenesis, treatment, and prognosis of the disease. Provided that the treatment is standardized, patients can live for many years as if they were normal people. This can assist patients in correcting their misconceptions about the disease and in reducing self-stigmatization. The behavior and communication of medical staff have a significant impact on patients.

### 4.2 Responses to stroke patients’ sickness stigma

The interviews highlighted the coexistence of both negative and positive coping mechanisms in response to illness stigma. Negative coping strategies and avoidance behaviors were evident in concerns regarding giving and receiving support from family, disruptions to one’s self-image, and denial of personal worth. Conversely, positive coping strategies were manifested through efforts to enhance self-worth via lifestyle improvements, active engagement in rehabilitation exercises, and fostering strong family and social support networks. Individuals tend to adopt non-adaptive emotion regulation strategies when they perceive negative events more intensely. Moreover, diverse individuals may respond to similar stressful situations in varied ways, and even the same individual may adopt different coping strategies based on their environment. Patients with higher social status and knowledge levels may initially resort to avoidance behaviors due to concerns about their public image. However, they may subsequently engage in positive adjustments to mitigate the impact of the disease on their personal image. Conversely, patients with lower levels of knowledge may feel a strong sense of duty towards their families post-recovery, but some may struggle with feelings of burden and opt for negative coping strategies. Effective psychological guidance, empathetic communication, and encouraging patients to establish correct values can help alleviate insecurities and mobilize motivation, thus facilitating recovery (Mohamad et al., 2023). Therefore, healthcare workers should engage in open communication with patients and offer psychological counseling services. For those who develop negative emotions due to their illness, professional psychologists can be arranged to provide guidance. Cognitive behavioral therapy can help patients identify and change irrational self-perceptions. Patients can also be organized to participate in support groups where they can share their experiences and feelings, gaining support from peers and enhancing their sense of self-worth. Narrative nursing can provide comfort and encourage patients to express their inner thoughts and emotions. This approach enables patients to release stress, enhance courage, and bolster confidence in confronting their illness.

### 4.3 Intervention strategies for stroke patients’ sense of shame

At the policy level, stroke patients suffer from physical disability and incapacity due to the disease, resulting in a huge economic burden to patients, their families and society. As such, economic conditions affect the stigma of the disease. By the end of 2022, the coverage rate of basic medical insurance in China remained above 95%, showcasing a trend towards comprehensive healthcare coverage across the nation. However, despite this achievement, there still exists a small segment of the population responsible for covering their own medical expenses. Moreover, due to the limitations of medical insurance, numerous medical items remain outside the scope of coverage, resulting in a significant economic burden on patients (Chinese Government Network, 2023). To address these challenges, it is recommended that medical insurance and relevant authorities consider expanding coverage to include commonly used drugs and nursing materials. Additionally, there is a need to enhance chronic disease treatment insurance and streamline the auditing process for chronic disease coverage. By lowering the threshold for chronic disease audit, individuals with chronic illnesses can access essential healthcare services and enjoy the associated life and economic benefits afforded by chronic disease insurance protection.

At the social level, while paying attention to the diagnosis and treatment of the disease, the dissemination of disease knowledge should also be promoted. Multi-channel and multi-form health education initiatives can enhance patients’ understanding of their condition, alleviate fear and irrational beliefs, and strengthen their confidence in treatment (McAleese et al., 2021). If the patient’s negative emotions are reduced and the compliance is improved, the patient is optimistic, the subjective initiative is improved, and the patient actively participates in rehabilitation training, which is conducive to the improvement of neurological function (Huang et al., 2021). When treating stroke patients, adopting a positive and optimistic approach can aid in diminishing feelings of shame, fostering a supportive social environment, raising awareness about the disease, and advocating for greater respect and care for stroke patients across society. Niemi et al. (1988) emphasized the importance of self-help programs and rehabilitation facilities to ensure that stroke patients receive the encouragement, psychological support, adaptative training, and adequate information on neuropsychological support they need. The robust development of the primary healthcare service system is crucial in transforming communities into key hubs for chronic disease prevention and treatment. Most stroke survivors reside in the community (Gillespie et al., 2020), by improving the service system of community hospitals and ensuring proximity to the public, making the community the main battlefield for chronic disease prevention and control, patients can receive comprehensive support to understand the pathogenesis, related risks, and effective prevention strategies of stroke. Furthermore, community-based initiatives aim to equip individuals with the necessary knowledge and skills to address stroke sequelae effectively, manage blood pressure, lipid levels, and blood glucose, and approach stroke events with a positive outlook.

At the institutional level, it is imperative to reinforce collaboration between medical institutions and community hospitals to facilitate the distribution of high-quality medical resources and address patients’ practical needs effectively. Medical personnel play a pivotal role in patient care, serving as primary conduits for disseminating disease knowledge, implementing treatment plans to alleviate suffering, and offering support to ease psychological distress. The attitudes of medical personnel towards patients have a profound impact on patients’ decision-making processes and emotional well-being, underscoring the importance of fostering positive and supportive interactions to guide patients towards optimal health outcomes. The role of medical staff is not only to treat patients, but also to provide good psychological guidance, help patients to correctly understand their own value, help patients to establish the concept of self-respect, reduce the uncertainty brought about by the disease, mobilize patients’ own motivation, and enhance the belief in disease recovery (Gillespie et al., 2020). Assisting patients in establishing short-term, medium-term, and long-term goals is crucial for facilitating progressive rehabilitation training. This approach enables patients to pursue rehabilitation incrementally, fostering the development of effective training habits. By breaking down goals into manageable steps, patients can maintain motivation and gradually progress towards their objectives. Moreover, setting goals helps redirect negative emotions, allowing patients to approach rehabilitation training with a positive mindset, which ultimately contributes to their recovery. Several scholars (Parry, 1996) have expressed the belief that individuals experience negative emotions after traumatic events because they cannot accept the changes to themselves. The emergence of negative emotions is intricately linked with cognitive processes. Enhancing irrational perceptions and modifying abnormal emotions and behaviors can enhance patients’ coping mechanisms, thereby mitigating the impact of negative emotions. Medical practitioners should exercise caution in their approach, fostering an environment where patients feel encouraged to open up about their experiences. It’s imperative to help patients recognize that their illness is but a fleeting episode in the broader spectrum of life, emphasizing the multitude of positive narratives that exist. Additionally, patients should be guided to accept physiological changes and adapt to new roles. Implementing evidence-based interventions to tackle the challenges posed by the disease is crucial, aiming for prompt recovery. By offering encouragement and support, enhancing patients’ understanding of their condition, and ensuring they feel respected and accomplished during the caregiving process, we can effectively stimulate their motivation, enhance their cooperation, facilitate disease rehabilitation, and alleviate negative emotions (Jia and Zhou, 2020). By using successful cases, effective communication abilities, and supplementary methods, the confidence of patients can be bolstered, facilitating their ability to surmount the ailment, redirect adverse emotions, and foster a constructive and robust mindset, thereby fostering patient compliance with diverse rehabilitation regimens (Xie and Wang, 2019).

At the level of family support, spousal support is important (Wang et al., 2020). Family is the main source of social support (Chang et al., 2022), and the understanding and support of spouses can reduce similar emotional and routine distress of patients. Good family support helps to improve the psychosocial outcomes of patients (McAleese et al., 2021). The lower the level of family support and social support the greater the stigma of illness (Wang et al., 2020). Stroke rehabilitation is a long process, and in this long-term process of seeking dependence, patients can be relatively sensitive to caregiver care emotions. The patients can perceive differential treatment from family and friends (Yang et al., 2019). The provision of care and support from both relatives and patients can engender feelings of warmth and esteem, ultimately enhancing treatment adherence and fostering subjective initiative, can mitigate negative emotions (Oliva-Moreno et al., 2018). Hence, family members ought to proactively engage in caregiving, aiding patients through the challenging phases of treatment and rehabilitation, establishing consistent dietary and lifestyle routines, and incorporating exercise and rehabilitation activities.

At the personal level, stroke patients are the ones who experience the shame of the disease, and there are many factors that influence patients’ sense of shame. Self-esteem is an individual’s self-emotional experience during social practice and reflects a general sense of self-worth and acceptance of one’s state. Appearance self-esteem represents the patient’s satisfaction with his appearance, behavioral self-esteem reflects the behavioral performance of the patient’s self-esteem, and social self-esteem refers to the patient’s desire for social evaluation (Xing, 2022). Stroke patients, typically elderly individuals, often possess a profound sense of familial and societal obligation. They maintain elevated expectations regarding their self-perception, and the repercussions of stroke inflict a substantial emotional burden. Consequently, they may exhibit pronounced emotional responses post-stroke, potentially culminating in psychological crises characterized by heightened anxiety and depression, thereby detrimentally impacting the patients’ overall health. When confronted with injury or challenging circumstances, individuals who actively seek social support exhibit a positive coping approach. In the context of disease onset and progression, the selection of coping strategies significantly influences the disease trajectory. The genesis of stroke-related shame is closely intertwined with the notion of preserving one’s social standing or “face” (Liu et al., 2023). If a disease affects the patient’s individual-social relationship, the limitation of the activity space will be more obvious, and the “dignity of the face” will bring greater pressure (Gong et al., 2023). Therefore, by relinquishing concerns about “face,” actively confronting the illness, rectifying detrimental habits, engaging in family activities, realizing personal worth, and dismantling societal stigmas, individuals can bolster their self-esteem. In daily rehabilitation and life activities, it is beneficial to guide patients towards adopting positive cognitive and emotional regulation techniques when faced with emotional challenges. Encouraging patients to perceive difficulties from alternative perspectives, fostering effective communication, and redirecting attention towards optimistic behaviors such as socializing, light aerobic exercises, or participating in manageable household tasks can contribute to their overall well-being. Those with low self-efficacy beliefs typically engage in more avoidance-based emotion regulation strategies and consequently report greater psychological distress (McAleese et al., 2021). Positive emotion regulation strategies can enhance patients’ psychological adaptation and their ability to confront the challenges of illness, alleviate negative emotions, and foster recovery of social functioning. It is recommended that medical staff adopt cognitive emotion regulation as a focal point for developing tailored intervention strategies to mitigate stigma and enhance patients’ empathy.

### 4.4 Strength and limitations

To maintain focus on the study’s objectives, interviews were conducted utilizing a semi-structured format. Interviewers encouraged patients to openly discuss their experiences of shame and coping mechanisms following a stroke through open-ended questions. This approach placed significant demands on the interviewers, who had to adeptly navigate patient dialogue. The aim was to investigate various coping strategies among stroke patients experiencing stigma. Additionally, it proposed methods to alleviate patient stigma through the perspectives of patients, caregivers, medical personnel, and policy frameworks. Notably, no qualitative studies on stroke-related stigma and coping were reported in China. By merging these two areas, the present study enriches and expands the existing research landscape. Employing qualitative research methods, particularly in-depth interviews with stroke patients, allowed for a comprehensive understanding of their coping strategies and underlying sentiments. This approach complements existing research on stroke stigma and equips medical professionals with insights to better assist patients and their families through psychological counseling and health education. By fostering a more comprehensive understanding of the disease and empowering patients to confront negative emotions, this study aims to enhance patient well-being. An inherent strength of the present study lies in its integration of interviews with observational data. This method enables a nuanced portrayal of patients’ experiences, while ensuring a quiet, private, and undisturbed interview environment conducive to candid discussions.

The researchers are relatively new to qualitative research, potentially lacking deep expertise in refining topics and analyzing data. The present study, limited by its cross-sectional design involving 14 patients with first-onset strokes, could not capture the dynamic psychological experiences and coping strategies evolving over time. Participants were recruited solely from one institution, raising concerns about the generalizability of the findings to stroke patients in other settings and regions, thus introducing potential bias. Moreover, due to the sensitive nature of the topic, respondents might have struggled to provide honest and comprehensive answers. Additionally, the interviewed patients were those capable of articulating their discomfort, possibly skewing the findings away from the experiences of severely affected stroke patients. Although the aim was to attain rich and comprehensive data, it was noted that interview responses began to repeat, indicating data saturation. Future recommendations include increasing the number of cross-cultural replication studies to test whether our findings are applicable to different sociocultural norms.

## 5 Conclusion

Findings from the present study demonstrate stigma is common in the early stages of stroke survivors. Physical impairments and self-care disabilities resulting from stroke can exacerbate patients’ experiences of stigma. This, in turn, can impact individuals’ problem-solving approaches and life attitudes, thereby affecting their work and familial dynamics to varying extents. Recognizing stigma early on is paramount to prevent the development of erroneous beliefs and maladaptive coping responses following a stroke. Strategies aimed at reducing stigma should encompass personal, familial, societal, policy, medical institutional, and environmental dimensions. Medical professionals play a pivotal role in helping patients cultivate accurate perceptions of their condition and foster beliefs in their ability to overcome it. Encouraging patients to adopt positive coping strategies is essential in mitigating their experience of shame. Simultaneously, involving patients’ families throughout the rehabilitation process and harnessing both familial and social support networks can aid patients in navigating the stabilization phase smoothly and facilitating their recovery.

### 5.1 Relevance to clinical practice

The present study emphasizes the origins of stigma as well as the importance of effective communication between healthcare providers and patients. It advocates for providers to empathetically engage with patients, fostering a sense of value and understanding. By prioritizing psychological care, healthcare professionals can alleviate patients’ disease-related fears, enhance their treatment self-efficacy, and bolster compliance with rehabilitation regimens. Further, the study highlights the pivotal role of family and medical staff in providing ongoing support to stroke patients. Encouraging patients to adopt a positive outlook on the future and harnessing their familial responsibilities to set goals can empower patients to redefine their abilities and confront challenges. Multi-faceted health education initiatives, encompassing disease knowledge and comprehensive medical insurance information, are also recommended to enhance patients’ disease understanding, alleviate fears, and reduce economic pressures, thus bolstering treatment adherence. To optimize clinical outcomes, healthcare providers should integrate these insights into personalized interventions. Early establishment of robust social support systems and tailored healthcare plans, taking into account patients’ familial support and financial circumstances, is crucial. Additionally, rehabilitation prescriptions should align with patient preferences to maximize engagement. By incorporating these behavioral considerations, healthcare professionals can effectively support patients in maximizing the benefits of rehabilitation efforts, ultimately enhancing treatment outcomes and overall quality of life.

## Data Availability

The original contributions presented in the study are included in the article/supplementary material, further inquiries can be directed to the corresponding author.
